# Development of Microalgae Biodiesel: Current Status and Perspectives

**DOI:** 10.3390/microorganisms11010034

**Published:** 2022-12-22

**Authors:** Livia Marques Casanova, Leonardo Brantes Bacellar Mendes, Thamiris de Souza Corrêa, Ronaldo Bernardo da Silva, Rafael Richard Joao, Andrew Macrae, Alane Beatriz Vermelho

**Affiliations:** 1 Biotechnology Center-Bioinovar, Institute of Microbiology Paulo de Goes, Universidade Federal do Rio de Janeiro (UFRJ), Rio de Janeiro 21941-902, RJ, Brazil; 2 Centro de Pesquisa Leopoldo Miguez de Mello, Petrobrás, Rio de Janeiro 21941-915, RJ, Brazil; 3 Sustainable Biotechnology and Microbial Bioinformatics Laboratory, Institute of Microbiology Paulo de Goes, Universidade Federal do Rio de Janeiro (UFRJ), Rio de Janeiro 21941-902, RJ, Brazil

**Keywords:** microalgae, lipids, biofuel, biodiesel, large-scale biodiesel production

## Abstract

Microalgae are regarded as a promising source of biodiesel. In contrast with conventional crops currently used to produce commercial biodiesel, microalgae can be cultivated on non-arable land, besides having a higher growth rate and productivity. However, microalgal biodiesel is not yet regarded as economically competitive, compared to fossil fuels and crop-based biodiesel; therefore, it is not commercially produced. This review provides an overall perspective on technologies with the potential to increase efficiency and reduce the general costs of biodiesel production from microalgae. Opportunities and challenges for large-scale production are discussed. We present the current scenario of Brazilian research in the field and show a successful case in the research and development of microalgal biodiesel in open ponds by Petrobras. This publicly held Brazilian corporation has been investing in research in this sector for over a decade.

## 1. Introduction

The world’s energy expenditure is expected to increase by approximately 50% between 2018 and 2050 [1]. Fossil fuels, a non-renewable energy source, provide around 80% of all energy consumed worldwide [1,2]. Their use, however, leads to large emissions of greenhouse gases (GHGs), mainly CO_2_, which is a major contributor to global warming [3,4]. Since the Kyoto Protocol (1996) and the Paris Agreement (2015) and the last report from the Intergovernmental Panel on Climate Change (IPPC), it is evident that urgent action is necessary to change this scenario. These reports recognize the interdependence of climate, ecosystems, biodiversity, and human societies, and the impact of CO_2_ and other toxic gases on the planet. Consequently, the industrial sectors are looking for ecological solutions and green technologies to reduce these emissions, resulting in alternative and innovative solutions [5,6].

Biofuels are one of the main alternatives to fossil fuel exploitation [7,8]. These fuels, produced from biomass or waste feedstocks, have the advantages of renewability and a significantly reduced contribution to global warming. The main biofuels available are biodiesel and bioethanol [2,9,10]. Other alternative green solutions are biomethane and biohydrogen [11]. Biodiesel is produced from lipids mainly by transesterification reactions having oils as the starting material [12,13]. Projections show that in 2040, biodiesel will account for 70% of the growing demand for transport fuel [14]. An increase in commercial biodiesel is observed. This rise was propelled by the increasing demand for green energy alternatives and public policies in many countries, including the United States, Brazil, and European nations, the leading world biodiesel producers [14,15,16]. Commercial biodiesel is currently obtained from different oil crops, such as soybean, corn, sunflower, and oil palm [9]. One of the main concerns related to these biodiesel sources is the use of arable lands resulting in competition with other segments, such as bioethanol and agriculture/livestock feed/food production [17]. Moreover, the time of production, climatic dependence, and soil quality, among other factors, introduce significant variability in crop production. In addition, fertilizer application releases nitrous oxide, a potent greenhouse gas [18].

High lipid contents make microalgae a promising alternative for biodiesel production. Besides this, microalgae are the major source of oxygen on the planet, and their CO_2_ biosequestration by photosynthesis point to the biodiesel from microalgae as a promising carbon-neutral fuel. In this context, microalgae biomass is emerging as a source of biodiesel [18]. These microorganisms show higher growth rates and productivity in comparison with conventional crops. Moreover, they can be cultivated using wastewater, thus avoiding competition for freshwater and increasing sustainability [8,9,19]. An essential differential is the concept of biorefinery applied to microalgae cultures. After lipid extraction, microalgal biomass residues contain several bioproducts of high value. This concept decreases the impacts of costs and productivity. Several countries, including Brazil, are investing in the development of algal biotechnology [11]. However, there are bottlenecks to be overcome, such as expensive and energy-intensive cultivation, microbial contamination, and the biodiesel conversion processes. All these factors lead to a higher production cost, the major challenge for biodiesel production after scaling-up [20].

In this review, we aim to provide a perspective of biodiesel production from microalgae, with an emphasis on technologies with potential applications to increase efficiency and reduce the overall costs of the process. These strategies include methods for increasing microalgal lipid productivity and production within a biorefinery concept, which enables the exploitation of valuable bioproducts such as carotenoids and fertilizers, amongst others. Moreover, we will present the progress and prospects of microalgae biodiesel production in Brazil.

## 2. Microalgae for Biodiesel Production

Microalgae are a diverse group of prokaryotic and eukaryotic photosynthetic unicellular organisms. More than 50,000 microalgal species live in various environmental conditions, including water domains such as streams, rivers, lakes, oceans, and terrestrial ecosystems [21,22,23].

Cyanobacteria are prokaryotic microalgae (Cyanophyta), while eukaryotic microalgae include Bacillariophyta (diatoms), Cryptophyta (golden algae), Rhodophyta (red algae), Xanthophyta (yellow/green algae), and Chlorophyta (green algae), amongst others [22,24]. The latter is the most promising group for biodiesel production. The selection of species for this purpose comprises criteria such as growth rate, tolerance to different environmental conditions, harvesting facility, and, most importantly, the lipid content, which ranges from 2 to 85% of dry biomass, depending on the species/strain and cultivation conditions [25,26].

**Figure 1:**
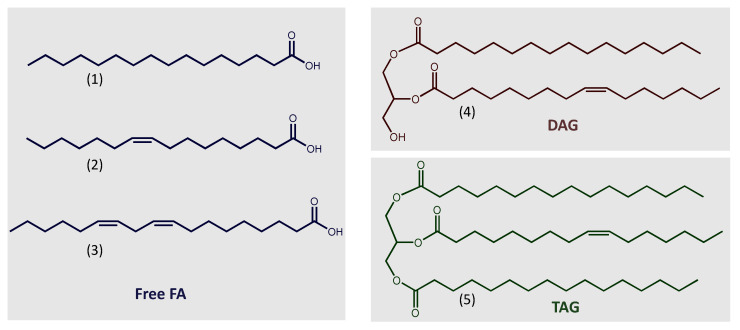
Chemical structures of commonly representative lipids. (**1**) Palmitic acid (16:0); (**2**) Palmitoleic acid (16:1); (**3**) Linoleic acid (18:2); (**4**) DAG (16:0/16:1); (**5**) TAG (16:0/16:1/16:0). Abbreviations: DAG, diacylglycerol; FA, fatty acid; TAG, triacylglycerol.

Microalgae, similar to other organisms, use neutral lipids for energy storage, while polar lipids are membrane constituents (Figure 1). They store acylglycerols, mostly triacylglycerols (TAG), during the day when photosynthesis occurs, and consume them at night to keep metabolic activities. TAG accumulation is induced by stress conditions, such as nutritional restriction, high temperature, and high salinity [25,27].

Microalgae can accumulate TAG to around 20–50% of their dry weight [9,25,28]. The fatty acids that constitute the acylglycerols in microalgae vary from C12:0 to C22:6. Qualitative and quantitative composition is diverse among different species. It is also highly dependent on nutritional and environmental conditions. However, most of their fatty acids have saturated and unsaturated C16 and C18 (Figure 1) carbon chains, such as palmitic (16:0), palmitoleic (16:1), oleic (18:1), linoleic (18:2), and linolenic (18:3) acids [22,25,29,30]. The fatty acid composition is relevant to the quality of the resulting biodiesel, influencing its outflow property, ignition quality, and oxidative stability [22,25,29].

## 3. Microalgal Production: Open X Closed Systems

The cultivation of microalgae can be carried out in open systems, closed photobioreactors, and, to a lesser extent, fermenters in the case of heterotrophic and mixotrophic conditions [31,32]. The first two systems are the most common and will be further discussed in this section.

### 3.1. Open Systems

In open cultivation systems, microalgae can be grown in lakes, lagoons, or ponds. Raceway shallow ponds are the most used for industrial purposes (food and cosmetic production). Open raceway ponds (OPR) are considered cost-effective, as they are cheaper to build, more straightforward to scale, and easier to operate when compared to photobioreactors. Another advantage is free sunlight energy [31,32,33,34].

On the other hand, open systems have many limitations, such as low biomass productivity, high harvesting costs, high water evaporation rates, and a high risk of contamination with other algae, bacteria, fungi, viruses, and predator protozoa species [31,32,33,34]. Additionally, open ponds suffer from poor CO_2_ mass transfer. Atmospheric CO_2_ usually does not meet the productivity requirements; thus, aeration and bubbling are necessary [31,34].

The lack of control over environmental factors affects productivity, which depends on weather conditions, photoperiod, and seasonal variations [31,34]. Therefore, choosing a location for microalgae cultivation in open systems is crucial, and must consider parameters such as solar radiation, pluviometric index, and local temperatures, besides land costs [34]. The most suitable locations for this cultivation are dry coastal areas in tropical and subtropical regions, with high solar irradiance throughout the year [35].

### 3.2. Closed Systems

Culturing in photobioreactors (PBRs) can overcome the limitations of open cultures. They enable a higher degree of process control and, consequently, higher productivity. PBRs have great versatility: several designs are available (e.g., tubular, flat plate, airlift, bubbling column), and various combinations of source light can be used, sunlight included [31,32,36]. In addition, they provide better CO_2_ utilization and maximum light exposure [31,37].

Despite the advantages, there is a significant limitation to using PBRs for large-scale production: the capital and operational costs [31,38,39]. The energy consumption by these systems is a major concern [31]. Pawar et al. [38] analyzed several economic models of biodiesel production costs in open raceway ponds and photobioreactors. The authors observed that the predicted costs of the latter are generally higher. Similar results were obtained in a more recent economic assessment, in which these two systems were compared for different geographic locations [40].

### 3.3. Hybrid Systems

As both open and closed culture systems have advantages and drawbacks, researchers proposed the combination of both for more cost-effective production. In a hybrid system, microalgae are grown in a PBR during the first stage, which favors high biomass productivity and minimum contamination. Subsequently, the produced biomass is transferred to OPR, aiming to achieve high lipid accumulation by applying stress conditions. Thus, the cultivation period in open ponds is shorter, and contaminants do not remain long enough to harm the culture [31,39,41,42].

Hybrid systems have shown higher lipid and biomass productivity when compared to open systems [41,42,43,44], and can therefore be considered promising for large-scale cultivation. However, techno-economic assessments are necessary to clarify their feasibility [42].

## 4. Biomass X Lipid Content: A Challenge

Despite the higher lipid yield of microalgae when compared to terrestrial crops, the overall costs to produce microalgal biodiesel are still high. Economic studies have pointed out lipid productivity as being a critical factor in enabling microalgal biodiesel to achieve favorable costs, compared with petroleum diesel [45,46,47].

Stress conditions generally induce lipid accumulation in microalgae. One of the most common strategies to enhance microalgal lipid content is limiting nutrients, mainly nitrogen, followed by phosphorus, sulfur, iron, and trace metals. Other stress conditions that affect lipid metabolism include high salinity and variations in the medium’s temperature, light, and pH [22,48,49]. The effect of these factors has been extensively reviewed elsewhere [22,25,49,50,51].

Nevertheless, stress conditions often negatively affect microalgal growth, resulting in lower biomass yield [48,52,53]. Due to these opposing traits, two situations are typical: high biomass production with low lipid content, and low biomass production with high lipid content. Both cases result in low lipid productivity [52,53,54]. Thus, finding conditions to induce high lipid accumulation, without interfering with growth and biomass production, is an important challenge for commercial biodiesel production [45,52,54].

The following section discusses methods proposed to increase lipid productivity based on two-stage cultivation, the addition of phytohormones and other chemicals, and co-cultivation. Approaches based on metabolic engineering and synthetic biology are also promising [49]. Key metabolic pathways in microalgae have been identified and are targets for manipulation to improve lipid productivity. Among the strategies evaluated, it is possible to highlight enhancing rate-limiting steps in TAG biosynthesis, blocking competing metabolic pathways (e.g., starch biosynthesis), reducing lipid catabolic pathways, and improving carbon fixation. Multitarget approaches, which enable the rational design of biological networks for the desired target products, are expected to produce the most encouraging outcomes. As a detailed discussion about these strategies is out of this review’s scope, the reader can refer to recent literature reviews to obtain further information [55,56,57,58].

## 5. Strategies to Increase Microalgal Lipid Productivity

### 5.1. Two Stage-Cultivation

Two stage-cultivation strategies aim to decouple biomass growth from lipid accumulation. In the first stage, microalgal growth is carried out under optimal conditions, while in the second stage, lipid accumulation is induced by applying stress conditions [42,48,53,54].

Nutrient starvation is the most frequent strategy to induce lipid accumulation [59]. Using a two-stage cultivation strategy for the species *Nannochloropsis oculata*, grown in nitrogen-sufficient conditions until the stationary phase and then transferred to a nitrogen-deficient medium, enabled lipid productivity almost 3-fold higher than with one-stage cultivation [60]. Similarly, the two-stage cultivation of *Chlorella* sp. with a nitrogen-starvation period resulted in higher biomass and lipid productivity [61]. Nayak et al. [62] recently reported a higher than 1.5-fold improvement in the lipid productivity of *Chlorella* sp. by employing this strategy.

Applying the two-stage cultivation strategy with nutrient restriction requires concentration of the biomass obtained in the first stage by harvesting, followed by transfer to a new culture medium [53,59]. The requirement of an intermediate harvesting step and transfer often demands more cost and energy [59]. Thus, an alternative to this issue is the development of strategies to eliminate these extra steps [53]. For instance, Amaral et al. [63] developed a method for two-stage cultivation in a modified PBR that eliminates harvesting before switching stages. Their device enables the circulation of the culture broth through the system by using a centrifugal pump. Stress promotion was performed by adding nutritional supplementation with nitrogen depletion when the stationary phase was reached. The method enabled a 55% increase in the lipid productivity of *Chlorella minutissima* [63].

Another possibility is the use of salt stress in a two-stage cultivation strategy. As salt can be directly added to the medium after the culture reaches the stationary phase, thus the extra harvesting and transfer steps can be eliminated, which contributes to the economic feasibility of the approach [53]. This strategy was successfully applied to *Scenedesmus obtusus*. Adding NaCl into the medium, when cultures reached the late exponential phase, resulted in lipid productivity 1.2 times higher [64]. With a similar strategy, it was possible to obtain promising results for *Desmodesmus abundans* [65]. The effect of NaCl in two-stage cultivation was also favorable to the lipid productivity of *Acutodesmus dimorphus*: a 43% increase in lipid accumulation was obtained without biomass reduction [66].

The combination of two or more stress conditions is also possible. Mirizadeh et al. [67] reported nitrogen and salt stress synergistic effects in a two-stage cultivation approach for achieving higher lipid productivity in *Chlorella vulgaris*.

### 5.2. Phytohormones Addition

**Figure 2:**
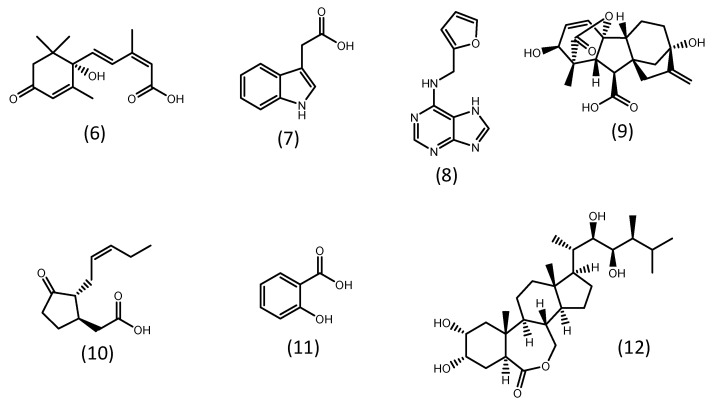
Examples of phytohormones. (**6**) abscisic acid; (**7**) indol-3-acetic acid (auxin); (**8**) kinetin (cytokinin); (**9**) gibberellic acid (gibberellin); (**10**) jasmonic acid (jasmonate); (**11**) salicylic acid; (**12**) brassinolide (brassinosteroid).

Phytohormones are small organic chemical messengers that play a broad spectrum of physiological roles in higher plants [68,69]. They are classified into five groups: auxins, abscisic acid, gibberellins, cytokinins, and ethylene. Other substances also act as phytohormones, including brassinosteroids, jasmonates, polyamines, salicylic acid, and signal peptides [69,70]. Some representatives of these groups are shown in Figure 2. The hormone systems of higher plants are likely to have evolved from a similar pre-existing system in microalgae. In fact, several phytohormones are known to be produced by microalgae. However, knowledge of their biosynthesis and physiological roles are still scarce [68,69,70,71].

The improvement in biomass production is often correlated with the increase in photosynthetic activity due to an increment in the expression of photosynthetic enzymes and chlorophyll content [69,71,72,73,74,75]. Concerning the accumulation of lipids, in several studies, the upregulation of enzymes related to the biosynthesis of fatty acids, such as acetyl-CoA carboxylase (ACCase), an acyl carrier protein (ACP), malonyl-CoA: ACP-transacylase (MCTK), and fatty acid desaturase (FAD) was reported [73,74,76,77,78].

It is generally observed that phytohormones enhance the adaptability of microalgae to biotic and abiotic stress conditions. Thus, phytohormones can aid microalgae in overcoming constrained biomass production under stress conditions, resulting in higher lipid productivity [69,70,71,72]. The combined effect of stress and phytohormones has been evaluated in some studies (Table 1) with positive results.

It is hypothesized that stress conditions inhibit cell growth due to the accumulation of reactive oxygen species (ROS). Phytohormones can aid the cells in keeping the ROS balance and reducing oxidative stress under these conditions by increasing the level of detoxifying enzymes and antioxidants [71,73,79,80].

The combined use of phytohormones on microalgae has also been assessed in some studies. Kozlova et al. [81] have evidenced the synergistic effects of 2,4-epibrassinolide (brassinosteroid) and indol-3-acetic acid (auxin) on cell growth and fatty acid accumulation of *Scenedesmus quadricauda*. A range of nano-concentrations of the combined phytohormones produced a 1.7-fold increase in cell density, and greater biomass and fatty acid production. These two phytohormones act synergistically in higher plants, and the cross-talk between their molecular pathways has been demonstrated [81].

Additionally, Seemashree et al. [82] reported that the combination of indol-3-acetic acid and amino ethyl hexanoate (DAH) enhanced the biomass and lipid production of two marine microalgae: *Porphyridium purpureum* and *Dunaliella salina*. Treatment with both phytohormones at the concentration range of 10^−7^ to 10^−8^ M resulted in a 2 to 3.5-fold increase in biomass, and up to a 1.4-fold increase in the lipid content of these microalgae [82].

**Table 1:** Effect of phytohormones on microalgae lipids and biomass.

Class	Phytohormone	Species	Stress Condition	Outcome	Reference
_	ABA	*Chlorella vulgaris*	None	Increase in biomass; 1.8-fold increase in lipid content	[83]
_	ABA	*Scenedesmus* *quadricauda*	N depletion	2.1-fold increase in biomass; little effect on lipid content	[84]
Auxins	DA	*Chlorella ellipsoidea*	None	7.1-fold increase in biomass; increase in overall lipid productivity	[80]
Auxins	DA	*Dunaliella tertiolecta*	Salt stress	40% biomass increase; lipid content increase from 24 to 70%	[85]
Auxins	DA	*Scenedesmus* *abundans*	None	5.4-fold increase in biomass; increase in overall lipid productivity	[80]
Auxins	IAA	*Chlorella* *sorokiniana*	N depletion	22% increase in biomass; 49% increase in lipid content	[73]
Auxins	IAA	*Chlorella* *sorokiniana*	N depletion	46% increase in biomass; 56% increase in lipid productivity	[74]
Auxins	IAA	*Chlorella vulgaris*	None	No effect on growth, 39% increase in lipid content	[77]
Auxins	IAA	*Nannochloropsis oceanica*	N depletion	1.5-fold increase in lipid productivity; decrease in SFA and increase in UFA content	[86]
Auxins	IAA	*Scenedesmus* *quadricauda*	None	Increase in cell growth, biomass, and fatty acid accumulation	[87]
Auxins	NAA	*Botryococcus braunii*	None	Increase in both lipid content and biomass	[76]
Brassinosteroid	EB	*Scenedesmus* *quadricauda*	None	Increase in cell growth, biomass, and fatty acid accumulation	[87]
Cytokinins	BAP	*Botryococcus braunii*	None	Increase in both lipid content and biomass	[76]
Cytokinins	KN	*Acutodesmus obliquus*	N depletion	50% increase in biomass productivity; 65% increase in lipid productivity	[72]
Cytokinins	KN	*Chlorella* *sorokiniana*	N depletion	36% increase in biomass; 52% increase in lipid productivity	[74]
Cytokinins	KN	*Desmodesmus* sp.	None	1.5-fold increase in growth rate; 2.5-fold increase in lipid productivity	[88]
Cytokinins	ZN	*Acutodesmus obliquus*	N depletion	61% increase in biomass productivity; 63% increase in lipid productivity	[72]
Ethylene	EP	*Botryococcus braunii*	None	No effect on biomass; increase in lipid content	[76]
Gibberellins	GA	*Botryococcus braunii*	None	No effect on biomass; increase in lipid content	[76]
Gibberellins	GA	*Chlorella ellipsoidea*	None	8.7-fold increase in biomass; increase in overall lipid productivity	[80]
Gibberellins	GA	*Chlorella* *sorokiniana*	N depletion	36% increase in biomass; 37% increase in lipid productivity	[74]
Gibberellins	GA	*Scenedesmus* *abundans*	None	5.3-fold increase in biomass; increase in overall lipid productivity	[80]
Jasmonates	JA	*Chlorella vulgaris*	None	51% increase in cell density; 54% increase in lipid content	[89]
Jasmonates	JA	*Chlorella vulgaris*	None	Increase in biomass; 2.1-fold increase in lipid content	[83]
Jasmonates	MJ	*Nannochloropsis oceanica*	None	Increase in biomass; 1.4-fold increase in lipid content	[79]
Others	DAH	*Chlorella* *sorokiniana*	N depletion	43% increase in biomass; 84% increase in lipid content	[73]
Others	DAH	*Desmodesmus* sp.	None	1.4-fold increase in growth rate; 2.5-fold increase in lipid productivity	[88]
Others	SA	*Chlorella vulgaris*	None	Increase in biomass; 1.7-fold increase in lipid content	[83]
Others	SA	*Nannochloropsis oceanica*	None	Increase in biomass; 2.2-fold increase in lipid content	[79]
Others	SA	*Phaeodactylum* *tricornutum*	None	No effect on growth; 29% increase in TGA content	[90]
Others	ST	*Monoraphidium* sp	None	Increase in biomass; 55% increase in lipid productivity	[78]

Note: In studies where various conditions were evaluated, the outcome reported in the table is the one with the best lipid and biomass productivity. Abbreviations: ABA = abscisic acid; BAP = 6-Benzylaminopurine; DA = 2,4-dichlorophenoxyacetic acid; DAH = diethyl aminoethyl hexanoate; EB = 2,4-epibrassinolide; EP = ethephon; GA = gibberellic acid; IAA = indole-3-acetic acid; JA = jasmonic acid; KN = kinetin; MJ = methyl jasmonate; N = nitrogen; NAA = 1-Naphthaleneacetic acid; SA = salicylic acid; SFA = saturated fatty acid; ST = strigolactone; TGA = triacylglycerols; UFA = unsaturated fatty acids; ZN = zeatin.

A combination of three hormones of different classes—zeatin (cytokinin), indol-3-acetic acid (auxin), and gibberellic acid (gibberellin)—was also able to increase biomass and lipid productivity of *Acutodesmus obliquus* under nitrogen limitation. These parameters were enhanced by 49% and 77% under the best conditions achieved [91].

### 5.3. Addition of Antioxidants and Other Bioactive Substances

**Figure 3:**
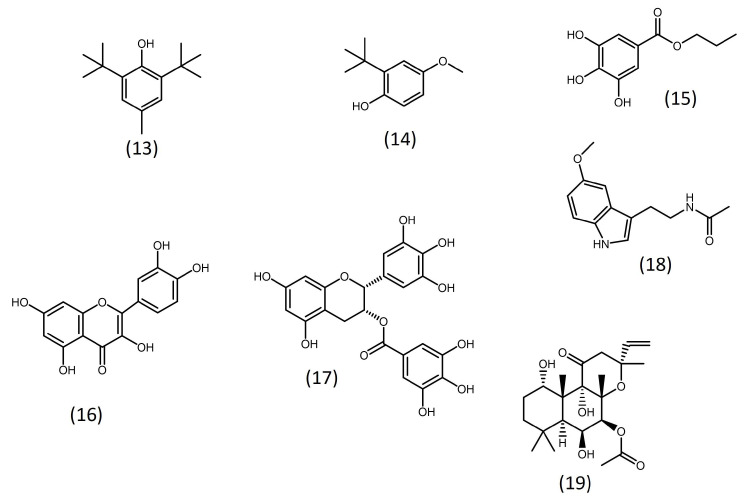
Structure of some chemicals with the potential to increase microalgal lipid productivity. (**13**) Butylated hydroxytoluene (BHT); (**14**) butylated hydroxyanisole (BHA); (**15**) propyl gallate; (**16**) quercetin; (**17**) (-) epigallocatechin gallate; (**18**) melatonin; (**19**) forskolin.

Some studies have evidenced that adding antioxidant substances (Figure 3) to culture media can enhance microalgal lipid productivity. This is the case for propyl gallate and butylated hydroxytoluene (BHT), two well-known antioxidants used in the food and pharmaceutical industries, which enhanced biomass and lipid productivities in *Schizochytrium* sp. [92].

Moreover, butylated hydroxyanisole (BHA) and propyl gallate, as well as the natural polyphenolic antioxidant (−)-epigallocatechin gallate, were shown to increase lipid accumulation in *Nannochloropsis salina* by up to 60% without negatively affecting growth [93]. Similarly, the plant polyphenolic quercetin increased the biomass productivity and lipid content of *Chlorella vulgaris* by 2.5-fold and 1.8-fold, respectively [94]. Phenolic compounds of natural origin, such as vanillin, *p*-coumaric acid, and lignoids, have beneficial effects on lipid microalgal productivity [95,96,97].

The effect of antioxidants on microalgal biomass and lipid content is not entirely understood. The over-production of ROS negatively affects photosynthesis, and can damage macromolecules, including lipids. Thus, antioxidants could reduce ROS levels and improve growth performance and lipid biosynthesis, especially under stress conditions, in which ROS levels are known to rise [71,93,98]. Other mechanisms may be involved as well. For instance, ethylenediamine tetraacetic acid (EDTA), an antioxidant and chelating agent, increased the lipid productivity of *Scenedesmus* sp. by 29.7%. EDTA can reduce oxidative stress and enhance the bioavailability of metal ions, such as iron and calcium, which are essential nutrients for microalgal growth and lipid production [99,100]. The effect of EDTA was also evaluated in *Chlorella ellipsoidea* and *Chlorococcum infusionum*. The substance increased lipid accumulation by 1.7-fold in the first specie, without an impact on biomass. In contrast, EDTA increased lipid content in the former species by 1.9-fold, positively affecting biomass [101].

Melatonin (Figure 3), another natural antioxidant, has also shown a positive effect on the lipid productivity of microalgae. It increased lipid accumulation of *Monoraphidium* sp. under normal and nitrogen-stress conditions by 1.2 to 1.4-fold, respectively [102,103]. The substance also increased the lipid productivity of *Monoraphidium* sp. under saline-induced stress [104]. Similarly, melatonin enhanced the biomass and lipid content of *Chlamydomonas reinhardtii* by 7.4 and 35.4%, respectively [98]. In both species, treatment with melatonin reduced oxidative stress [98,103,104]. However, there are other potential mechanisms through which this substance may exert the observed effects. Zhao et al. [102] showed that melatonin influences phytohormones produced by *Monoraphidium*, upregulating gibberellic acid, and downregulating abscisic acid. Additionally, the substance was shown to upregulate cell autophagic activity, a process related to oxidative stress response and lipid accumulation [102,104].

Besides antioxidants, some studies evaluated other compounds as potential modulators of lipid accumulation. Franz et al. [93] conducted a phenotypic screening with 54 commercially available substances to identify small molecules that are able to increase the growth and lipid accumulation of four microalgal strains (*Nannochloropsis salina*, *Nannochloropsis oculata*, *Nannochloris* sp., and *Phaeodactylum tricornutum*). Bioactive molecules such as forskolin (Figure 3), cyclic adenosine monophosphate (cAMP), orlistat, quinacrine, as well as previously mentioned common antioxidants, were among the compounds considered most promising [93].

In a high throughput screening approach, a library of 43,783 synthetic compounds was evaluated in *Chlamydomonas reinhardtii*. Fifteen of them, classified into five groups according to structural similarity, were considered high performing in terms of enhancing lipid content and growth. Representative substances of each group were also evaluated in three additional microalgal species (*Chlorella sorokiniana*, *Chlorella vulgaris*, and *Tetrachlorella alterens*) and have shown similar results, confirming their potential to be employed in biofuel production [105].

### 5.4. Co-Cultivation

In nature, microorganisms are found in complex and dynamic communities. Microalgae are not different; they live symbiotically with associated bacteria and fungi [106,107]. Synergistic interactions among microorganisms have been extensively used for industrial processes, such as soil remediation, wastewater treatment, and food production. Thus, not surprisingly, co-cultures of microalgae with other microorganisms can potentially increase biomass and lipid productivity [107,108,109,110,111].

Among the possible strategies of this kind, the co-culturing of microalgae and oleaginous yeasts is one of the most studied. In this cultivation system, the microalgae provide O_2_ to the yeast, while the former provides CO_2_ to the microalgae. Moreover, organic acids produced by the yeast, which can inhibit its growth, can be taken up by the microalgae. The yeast can also metabolize complex sugars into simpler ones to be taken up by the microalgae. Another advantage of the association is the pH balance. While microalgae in monoculture tend to make the medium more alkaline, yeasts grown alone make the medium more acidic, which is detrimental to their growth. Together, they can keep the pH in an optimal range for both [107,108]. For instance, the co-cultivation of the microalgae *Chlorella pyrenoidosa* and the red yeast *Rhodotorula glutinis* in cassava bagasse hydrolysate reached a significantly higher biomass and lipid productivity when compared to monocultures [112]. Another study evidenced that the co-encapsulation of *Chlorella vulgaris* and the yeast *Trichosporonoides spathulate* leads to high biomass and lipid productivity, with the advantage of facilitating the subsequent harvesting process [113]. More examples of microalgae and yeast co-cultivation systems can be found in recent reviews [107,108].

Mutualistic interactions between microalgae and bacteria are also documented. Bacteria produce important substances for microalgal development, such as macronutrients, vitamins, siderophores, and growth stimulants [106,110,114]. Some studies reported the benefits of microalgae and bacteria co-cultivation for improving algal lipid productivity. For instance, Toyama et al. [109] observed that the co-cultivation of *Euglena gracilis* with the bacterium *Emticicia* sp. EG3 in wastewater enhanced the microalgae’s biomass and lipid content by 3.2 and 2.9-fold, respectively. Additionally, a recent study showed that the co-cultivation of actinomycetes, bacteria known to produce microalgae-stimulant growth factors, and the Chlorophyta *Tetradesmus obliquus*, grown in wastewater, enhanced its lipid productivity by 1.55-fold [115]. Co-cultivation systems with bacteria can also improve and reduce harvesting costs by promoting cell bioflocculation [116,117].

Co-cultivation systems, composed of different microalgal species, are another possibility [111,118,119]. Stockenreiter and Litchman [118] evaluated the co-cultivation of Chlorophyta and Cyanobacteria species, two major microalgal taxa with different functional characteristics, such as nutrient requirements and light wavelength absorption. The authors studied systems composed of two, four, and eight species from both groups. They observed that the growth and lipid productivity increased with increasing diversity; cultures with a higher number of species showed higher productivity. Lipid yields were notably higher in cultures containing *Anabaena cylindrica*, nitrogen-fixing cyanobacteria. They hypothesize that the complementary traits between distinct microalgal groups can provide a more efficient use of resources [118].

## 6. Large-Scale Production: Current Scenario and Perspectives

As previously stated, microalgal biodiesel production is in its infancy and has not yet achieved the commercial stage. Even though most of the research on the subject has been carried out on a laboratory scale, some recent reports on pilot-scale studies and scale-up experiments are available [120,121,122,123,124,125,126,127,128,129,130,131].

An important challenge for the viability of commercial microalgal biodiesel production is the lower biomass and lipid productivity of large-scale outdoor microalgae cultivation [129]. For instance, Lu et al. [132] observed that the biomass productivity of *Chlorella* sp. in an outdoor PBR was more than 50% lower compared to bench-scale indoor conditions, while the lipid productivity was three times lower. However, lower yields are not a rule for large-scale cultivation; the biomass and lipid productivity of *Ascochloris* sp., cultivated in pilot-scale outdoor open troughs, did not significantly differ from those obtained with bench-scale indoor PBR [126].

Most of the pilot scale studies carried out with microalgae for biodiesel production do not focus on economic aspect analysis. Thus, even though the technical viability of the large-scale production is demonstrated, little is known about their economic feasibility. According to Chisti [133], to have competitive prices relative to petroleum, algal biomass must be produced at around US$0.25/kg (dry weight). The biomass production of *Synechocystis* sp. in a large-scale cultivation OPR system was estimated to be approximately US$2–3/kg [121]. Similarly, the biomass cost of *Scenedesmus acuminatus* in OPR was predicted to be US$1.76/kg. On the other hand, the biomass production of the same species in PRB was calculated to be US$6.91/kg [134]. In another report, the biomass cost of *Chlorella vulgaris* in a pilot-scale outdoor PBR was estimated at US$14.3/kg [130]. These results are close to theoretical estimates, which projected the costs of biomass production in OPR to be around US$0.4–2.1/kg (€0.3–1.8) and in PRB around US$4.5–11.8/kg (€3.8–10) [135], confirming that production costs in OPRs are lower than in PBRs. However, the current costs are still far from the desirable condition, which evidences the need for optimization and technological improvement in the process.

## 7. Biorefinery Concept and Other Strategies to Reduce Production Costs

A promising possibility to enhance the economic competitiveness of microalgal biodiesel is production within a biorefinery concept. In a biorefinery, the biodiesel production from lipids would be integrated with the simultaneous production of other high value biomass components. Besides lipids, microalgae contain carbohydrates, proteins, and a variety of complex organic molecules such as vitamins, terpenes, and carotenoids. These components can be extracted and transformed into a variety of by-products, such as bioethanol, biogas, chemicals, food supplements, animal feed, fertilizers, cosmetics, and nutraceuticals [136,137,138].

The aggregation of other valuable goods to the biodiesel production chain allows better material and energy utilization, enables greater product flexibility, reduces the generation of residues, and thus improves the feasibility of the whole process [136,137,138]. Branco-Vieira et al. [138] analyzed possible configurations for a biorefinery to use the biomass from *Phaeodactylum tricornutum*. They assessed the chemical composition of the biomass cultivated in pilot-scale outdoor PBR, and used these data to build scaled-up scenarios. The authors concluded that integrating biodiesel, bioethanol, fucoxanthin, and biosilica production would be feasible [138].

Some techno-economic evaluations have shown that biodiesel production in integrated biorefineries is more economically and environmentally favorable [136,139]. However, in the case of biodiesel production coupled with high-value products, there is a concern that the increased production of the latter may cause market saturation, as the demand for such products is not high [136,140]. Moreover, concerning food components, safety issues may arise when their production is integrated with that of fuels and chemicals [141]. Due to these concerns, Lee et al. [140] favor an integrated production of various biofuels.

Another way to improve the economic feasibility of microalgal biodiesel is by reducing nutrient supply costs. Currently, large-scale microalgal cultivation uses commercial CO_2_ and agricultural fertilizers. It is, however, possible to project cultivation systems in which the CO_2_ is obtained from flue gases emitted by industries and/or in which nitrogen, phosphorus, and other nutrients are obtained from wastewater. These approaches can potentially reduce costs and the environmental impact of the process [133,136,142].

Selvan et al. [143] demonstrated biodiesel and bioethanol production by *Acutodesmus obliquus* cultivated in cassava wastewater in an OPR pilot-scale system. In another study, the pilot-scale cultivation of *Ascochloris* sp. in an open system with dairy wastewater achieved high biomass and lipid content of almost 35% [126]. Recently, a cultivation system for *Scenedesmus quadriculata* with turbulence pulsation and phytohormones (gibberellic acid, indole-3-acetic acid, and brassinolide) evidenced high recovery rates and lipid productivity [144]. Moreover, Park et al. [145] evaluated the cultivation of *Nephroselmis* sp. in pilot-scale PBR using flue gases from a thermal power plant as a CO_2_ source, evidencing the process as feasible for large-scale cultivation. The use of flue gases also showed promise for the large-scale cultivation of *Nannochloropsis* sp in OPR [146]. Nayak et al. [142] demonstrated the suitability of cultivating *Scenedesmus* sp. with domestic wastewater and CO_2_ from flue gas in both PBR and OPR.

In summary, possible ways to improve the commercial feasibility of microalgal biodiesel are:

(a). Development of new technologies to increase lipid and biomass productivity of large-scale cultivation, as well as to reduce the operational costs of the process;
(b). Integration of the biodiesel production with other biofuels or added-value by-products;
(c). Use of low-cost nutrients from wastewater and CO_2_ from flue gases.

Nonetheless, it is worth pointing out that economic competitiveness is not the only important factor for microalgal biodiesel to achieve marketability in the global fuel scenario. Public policies have always played a significant role in the development of the biodiesel industry throughout the world. Policies introduced in the United States, European Union countries, and South American countries (majorly Brazil and Argentina) have been the driving force for the development of the biodiesel industry and market in these nations. Public support strategies include financial support for research in the field, price subsidies, blending mandates, and quantitative targets [14,147]. For instance, in Brazil, the National Agency for Petroleum, Natural Gas and Biofuels (ANP) establishes a mandatory biodiesel fraction in commercial diesel, which has gradually increased since 2008 reaching 13% [148,149].

The biodiesel demand will likely increase in the coming years, driven by the growing world energy demand combined with the urgent need for green energy alternatives to mitigate climate change. Therefore, microalgal biodiesel is still promising and likely to attract future government support. 

## 8. Biodiesel from Microalgae in Brazil

The Brazilian government started to invest in biodiesel in 2004 with the creation of the National Program for the Production and Use of Biodiesel (PNPB), whose objective was to stimulate biodiesel production in the country. New technology routes and research development were established and Brazil, together with the USA and Indonesia, are the major world producers and consumers of biodiesel. The biodiesel currently produced in Brazil is mainly obtained from animal fats and vegetable oils (1G biodiesel) [150]. The most common production process has been the chemical route by the transesterification of TAGs with methanol or other short-chain alcohols, through homogeneous alkaline catalysis with hydroxides (e.g., NaOH and KOH) or alkoxides (e.g., CH_3_O^−^ Na^+^ and CH_3_O^−^ K^+^). The alternative enzymatic route is ecologically friendly, uses less energy and water, and produces purer glycerol. However, it is a more expensive method [151]. Therefore, the search for sustainable processes turns to the enzymatic route, a biotechnological process in progress [152].

Microalgae biomass has received attention for third-generation biofuel production due to its high carbohydrate and TAG content. These microorganisms are attracting renewed attention due to the possibility of using their carbohydrates for bioethanol, and lipids for biodiesel production. However, their use has yet to achieve full industrial scale [153].

The Brazilian government started to invest in microalgae research in 2008 in a joint action with other public agencies such as the Secretariat of Aquaculture and Fisheries (Secretaria de Aquicultura e Pesca) and *Conselho Nacional de Desenvolvimento Científico e Tecnológico*—CNPq). As a result, a fund was opened for research focused on biodiesel production from microalgae. In 2013 a new governmental investment was made with the partnership of CNPq for biofuels and bioproducts from microalgae [11]. Since these investments, microalgae research in Brazil has been developing and mobilizing several public institutions and academic laboratories. Consequently, many partnerships have been formed between companies and universities. An increased interest in renewable energy has arisen, and the research concerning biofuels has intensified.

Several microalgae were isolated from Brazilian biomes, such as the Amazon Forest, the Cerrado, and the Pantanal flooded grasslands. In addition, microalgae were isolated from wastewater deposits generated by industrial and agricultural activities such as pisciculture, the sugarcane industry, and swine farming. The identified specimens were deposited in specific collection of microorganisms for agro-energy and biorefineries maintained by the Brazilian Agricultural Research Corporation (Embrapa) [154].

Brazilian scientific articles describe the research for microalgae focusing on biodiesel production. Microalgal Brazilian biodiversity is very significant. For instance, *Botryococcus* found in Brazil, is a microalgae genus rich in monounsaturated fatty acids, and is an excellent candidate for biodiesel production. The authors demonstrated that nutrient manipulation could stimulate the production of different compounds [155].

Ribeiro et al. [156] isolated six microalgae from the Midwest Region of Brazil (Mato Grosso do Sul, Dourados) cultivated in open ponds with potential use for biodiesel production. They observed that *Pseudokirchneriella subcapitata* presented high growth, while *Coelastrum* sp. and *P. subcapitata* showed the highest lipid contents, corresponding to approximately 20% of their dry mass [156]. In a study by Calixto et al. [157], it was demonstrated that the microalgae *Chlorella* sp. (D101Z), *Chlamydomonas* sp. (D132WC), *Pediastrum tetra* (D121WC), *Scenedesmus acuminatus* (D115WC), and *Sinechocystis* sp. (M3C) are promising candidates for biodiesel production [157]. A method for disrupting the cell wall by non-thermal plasma was carried out with *Nannochloropsis gaditana* to improve lipid extraction [158].

The green microalgae *Monoraphidium contortum* (CCMA-UFSCar701), isolated from freshwater in mangrove areas of Central and North Coasts of São Paulo State, Brazil, presented a high lipid content (43.60%) close to that observed in *Botryococcus braunii* (UTEX-2441; 48.85%), a strain from UTEX Culture Collection (USA) [159].The genus *Scenedesmus* sp. has been a target for biodiesel research [160]. Several authors focused on microalgal biodiesel using *Scenedesmus* sp and agro-industrial wastewater [161] or agro-industrial effluents [162]. The microalgae *Chlorella protothecoides* demonstrated excellent properties for biodiesel [163].

Brazilian companies, such as Petrobras (Petróleo Brasileiro S.A, Rio de Janeiro, Brazil) and Embrapa (Brasília, Brazil) have invested in microalgae research in recent years. A Program, created by Embrapa, isolated, identified, and evaluated biotechnologically important microalgae species in Brazil, integrating the biorefinery concept into the biofuels [164].

Another association of the Federal University of São Carlos and the company Algae Biotecnologia received a fund from the national development bank (BNDES) to develop a project of a biodiesel pilot plant, using vinasse for microalgae digestion and cultivation [153].

Below, we show a successful case in the research of biodiesel from microalgae in Brazil, which is the project from Petrobras. It is still under development but has several stages already established.

## 9. Petrobras’s Microalgae Biodiesel Project 

Petrobras has invested in partnerships with public research universities to develop microalgae research since 2009. In this context, this integrated approach was successfully applied in a project developed by Petrobras, in cooperation with the State University of Campinas (UNICAMP), the Federal University of Viçosa (UFV), the Federal University of Rio de Janeiro (UFRJ), the Federal University of Rio Grande (FURG), and the Federal University of Rio Grande do Norte (UFRN). Petrobras established a cooperation with the last institution to develop an open ponds pilot plant to test microalgae for biodiesel production [165].

Scaling up for biodiesel production is an integrated process including the choice of microalgae, culture medium, harvesting of microalgae, oil extraction, and conversion to biodiesel. All these factors are relevant to large-scale upstream production. The microalgae used in the process must present specific properties, including resistance to biological contamination and high lipid productivity. The phases of the upstream process, starting with the inoculation, are responsible for approximately 70% of the costs involved in the generation of biodiesel from microalgae. In this stage, genetic modification of microalgae by synthetic biology to increase the biosynthesis of target molecules is an excellent strategy. Recently, the joint venture formed by the companies ExxonMobil and Synthetic Genomics in the USA used this approach [166].

Cell disruption followed by oil extraction is a critical point in the process, and needs to be standardized according to the conditions of the bioprocess to obtain the best oil yield. However, other selection criteria for microalgae must be considered if the bioprocess is coupled with the biorefinery concept, according to the desirable bioproducts derived from the biorefining processes (downstream).

One of the challenges of the project was the resistance to cell disruption. This problem led the group to reevaluate microalgae management and select the species used in open biomass production systems (open pond). The biomass generated was adjusted to meet the requirements found throughout the bio-refining process. The process was successfully subjected to a cold oil extraction technique, developed jointly with the Federal University of Viçosa, for later conversion to biodiesel with low energy consumption and recovery of the solvents used.

**Figure 4:**
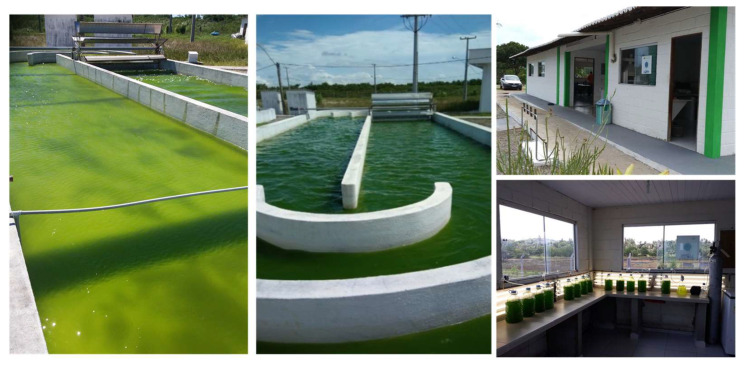
Petrobras pilot-scale open pond microalgae production facility in Rio Grande do Norte (Brazil).

In Rio Grande do Norte, the scale-up was successful (Figure 4) and reached high biomass production using native microalgal strains. The pilot plant has open ponds with a total area of 100 m^2^, producing 6000 L of algae and a yearly productivity of 30 g/m^2^/day. These results at this scale indicate that an integrated biorefinery approach is likely to achieve cost effective microalgae biodiesel. 

Petrobras in partnership with universites has overcome many of the scale-up challenges, and solutions for other challenges are in development. In this context, integrating the results obtained in the cultivation process and refining the collected biomass (bio-refining) must be considered an essential factor for developing the product “microalgae biodiesel”.

In conclusion, the future of microalgae biodiesel is bright and increasingly within a circular economic framework where waste residues from one industry serve as inputs to its production and where algae not only produce biodiesel but other bioproducts too. The improved economics of the scale-up process is in progress, and commercial production will be possible within a low-carbon economy shortly.
